# Highly bendable bilayer-type photo-actuators comprising of reduced graphene oxide dispersed in hydrogels

**DOI:** 10.1038/srep20921

**Published:** 2016-02-11

**Authors:** Dowan Kim, Heon Sang Lee, Jinhwan Yoon

**Affiliations:** 1 Department of Chemistry, 37 Nakdong-Daero 550beon-gil, Saha-gu, Busan 49315, Republic of Korea; 2 Department of Chemical Engineering Dong-A University, 37 Nakdong-Daero 550beon-gil, Saha-gu, Busan 49315, Republic of Korea

## Abstract

To avoid the problem of reduced graphene oxide (rGO) restacking in aqueous solution, the preparation of light-responsive poly(*N*-isopropylacrylamide) incorporating rGO (PNIPAm/rGO) was achieved by the chemical reduction of GO dispersed in the hydrogel matrix. Due to the enhanced photothermal efficiency of the rGO, the prepared PNIPAm/rGO underwent large volume reductions in response to irradiation by visible light of modest intensity. With respect to potential applications, bilayer-type photo-actuators comprising a PNIPAm/rGO active layer and poly(acrylamide) passive layer were fabricated; these achieved a full bending motion upon visible-light exposure. Adjusting the swelling ratio of each layer in the initial state yielded bidirectional photo-actuators that showed the active motion of turning inside out. Furthermore, we demonstrated that the fabricated actuation system would exhibit controlled bending motion in response to solar radiation.

Three-dimensional micro-actuators comprising soft hydrogels have received a great deal of attention because of their potential applications in biomedicine^1^,^2^, robotics^3^,^4^,^5^,^6^,^7^,^8^,^9^,^10^,^11^,^12^,^13^, and microfluidics^14^,^15^,^16^,^17^. Local or global changes in the volume or shape resulted from the swelling of the hydrogels can induce the mechanical movement including bending and twisting. Since stimuli-responsive hydrogels can undergo desirable volume changes in response to external stimuli such as heat^3^,^4^,^ ^pH^5^,^6^, ion strength^7^, light^8^,^9^,^10^,^11^,^12^,^13^,^14^,^15^,^16^,^17^,^18^,^19^,^20^,^21^,^22^, and magnetic/electric field^23^,^24^, they can serve as platforms for soft actuators that can perform mechanical actions on demand. Much research effort has been devoted to the development of smart actuation systems employing light-responsive hydrogels^8^,^9^,^10^,^11^,^12^,^13^,^14^,^15^,^16^,^17^,^18^,^19^,^20^,^21^,^22^, since light would be an attractive stimulus for manipulating the actuators, enabling rapid on-off switching, remote control with variable intensity, and independent local control.

Thermally responsive hydrogels containing photothermal materials have provided efficient platforms for controlling the volumes or shapes of hydrogels in response to visible light or near infrared radiation^8^,^9^,^10^,^11^,^12^,^13^,^14^,^15^,^16^,^17^,^18^,^19^,^20^,^21^,^22^. Graphene oxide (GO), in particular, would be an attractive photothermal material for such hydrogels because of its good water dispersibility^25^ and high compatibility with biomolecules^26^. It has been reported that GO, when dispersed in a thermally responsive hydrogel matrix, converted photo energy to thermal energy, thus providing sufficient heat to shrink the hydrogel^15^,^16^,^21^,^22^. To be utilized widely, a large volume change by the hydrogel in response to light irradiation of modest intensity is typically required. Even though a higher concentration of the GO can induce greater volume changes in the composite hydrogels, they have been reported to aggregate in the hydrogel matrix above a critical concentration, which would disturb the homogeneous hydrogel structure^27^. Consequently, an effective way to improve the light-induced volume shrinkage would be to enhance the photothermal efficiency of the GO.

Motivated by the enhanced photothermal efficiency of reduced graphene oxide (rGO) reported in recent studies^28^, we pursued the preparation of composite hydrogels containing rGO to maximize the light-induced volume shrinkage. However, polymerization of the monomer solution containing rGO was not feasible due to the aggregation of rGO. Since the oxygen containing group located in the planar basal plane of GO removed during the reduction reaction, rGO is prone to restacking due to strong π-π interactions and van der Waals forces between the exfoliated sheets^25^. Even though surfactants and polymers have been used as effective stabilizers to prevent aggregation during reduction, these methods require additional, inconvenient chemical reactions^29^,^30^. Furthermore, photothermal efficiency of surfactant-coated graphene is lower than rGO.

In this work, to prepare well-dispersed rGO-loaded hydrogels while avoiding the restacking problem, we attempted reduction of the GO sheets after their fixation in the hydrogel network through gelation of the monomer solution containing GO. Based on the improved photothermal efficiency of the rGO, we fabricated a light-driven bilayer-type actuator that show large bending motion.

## Results and Discussion

**Figure 1:**
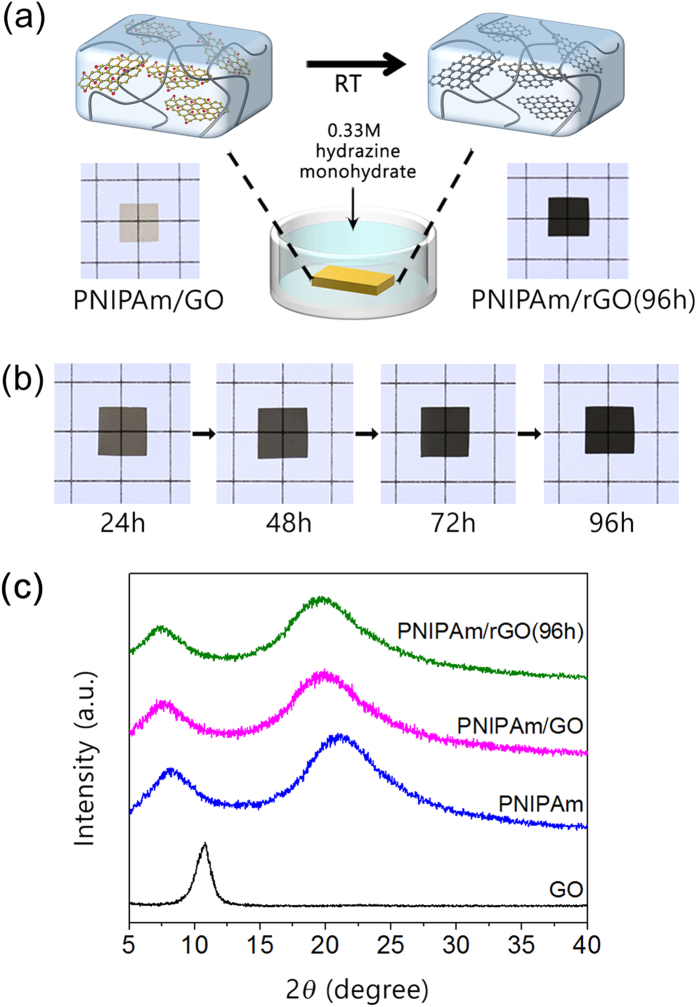
(**a**) Schematic illustration for the reduction of graphene oxide (GO) dispersed in the hydrogel. The PNIPAm/GO composites immersed in aqueous hydrazine monohydrate for time *N* are designated as PNIPAm/rGO(*N*). (**b**) Photographs of poly(*N*-isopropylacrylamide)/reduced graphene oxide (PNIPAm/rGO) hydrogels after various reduction time. (**c**) XRD profiles for GO, PNIPAm, and PNIPAm containing 3.3 wt% GO (PNIPAm/GO) before and after reduction in hydrazine monohydrate solution for 96 hours (PNIPAm/rGO(96 h)).

Our strategy for the reduction of the GO dispersed in the hydrogel matrix is illustrated in Fig. 1a. First, the hydrogel composites were prepared from the thermally responsive monomer *N*-isopropylacrylamide (NIPAm), the cross-linker *N,N*′-methylenebis(acrylamide) (BisAA), and an aqueous GO dispersion through a chemically initiated free-radical polymerization^21^. The reduction of GO dispersed in the hydrogel matrix could be achieved by immersing the hydrogel composite in 0.33 M aqueous hydrazine monohydrate at RT. Hydrazine monohydrate is most frequently used chemical reductant to yield highly reduced GO through simple procedure. In this approach, hydrazine monohydrate solution is diffused into the hydrogel matrix by swelling, resulting in the reduction of the GO dispersed in the network without any restacking problems. Even though the effective reduction time can be shortened by increasing temperature, medium temperature was adjusted at RT to prevent the aggregation or degradation of poly(*N*-isopropylacryamide) (PNIPAm). The PNIPAm/GO composites immersed in aqueous hydrazine monohydrate for time *N* are designated as PNIPAm/rGO(*N*). As shown in the photographs in Fig. 1b, we found that the color of the composite hydrogel changed from transparent brown to transparent black after the reaction with hydrazine monohydrate. This color change indicates that the GO sheets entrapped in the hydrogel matrix were reduced without forming aggregated domains, since the change is directly related to the decrease in oxygen-containing function groups on the surface of the sheets^31^. We also reveal that the black color of the composite is get darker as increase of reaction time, indicating that color change can be an obvious visible indicator of the effect of reduction. We note that electrochemical or chemical reduction with sodium ascorbate at high temperature have recently been used to prepare the rGO-based hydrogels^32^,^33^.

X-ray diffraction (XRD) experiments were conducted to determine that rGO is indeed present as individual sheet in the hydrogel matrix^34^. Figure 1c shows the XRD profiles for pure GO and PNIPAm containing GO before and after reduction in hydrazine monohydrate solution for 96 h. The characteristic diffraction peak of GO was observed at 2*θ* = 10.8°, corresponding to the interlayer spacing of stacked GO sheets, After GO was dispersed into the PNIPAm matrix through polymerization, the XRD profile for the composite showed only two broad peaks corresponding to the PNIPAm diffraction pattern. The typical GO peak completely disappeared, suggesting that the exfoliated GO sheets were well-dispersed in the PNIPAm matrix. Even after reduction of GO dispersed in PNIPAm, only characteristic peaks for PNIPAm were observed, confirming that rGO sheets were dispersed well in the hydrogel and were exfoliated.

**Figure 2:**
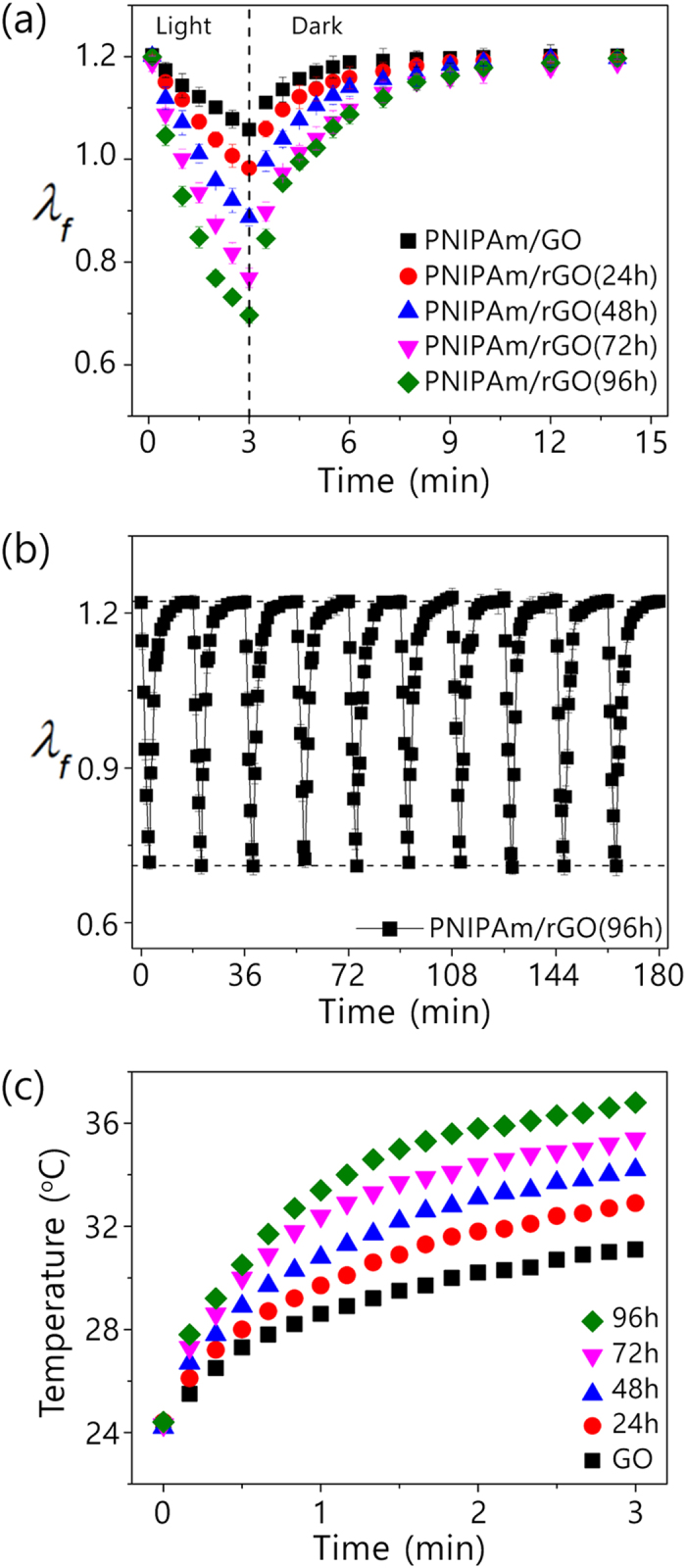
(**a**) Linear swelling ratio *λ*_*f*_ for PNIPAm/GO and PNIPAm/rGO(*N*) exposed to blue light at 41.8 mW/cm^2^ for 3 min. (**b**) Linear swelling ratio *λ*_*f*_ for PNIPAm/rGO(96 h). Each cycle consists of blue light illumination for 3 min followed by a recovery time of 15 min. (**c**) Temperature changes for 2.5 mg/mL aqueous GO and rGO of various reduction times under blue-light irradiation.

To investigate the photothermal efficiency of the rGO obtained by the above approach, the light-induced volume change of the hydrogel composites was measured using visible light from a high-pressure mercury short arc lamp with a wavelength of 450–490 nm and intensity of 41.8 mW/cm^2^. As shown in Fig. 2a, the PNIPAm/rGO retains nearly the same linear swelling ratio (*λ*_*f*_) of 1.20 at equilibrium as PNIPAm/GO regardless of reaction time. This result reveals that the reduction of GO dispersed in the hydrogel network have little influence on the swelling behavior of the composite hydrogel. The equilibrium linear swelling ratio, *λ*_*f*_, is defined as the amount a gel swells in each dimension at equilibrium state when immersed in water. When exposed to blue light, the hydrogel containing GO or rGO underwent significant volume changes depending on the chemical state of GO. As can be seen in Fig. 2a, the degree of *λ*_*f*_ change induced by light irradiation ranged from 18.3% to 41.7% after 3 min exposure in proportion to the reaction time (from 24 to 96 h), whereas that of PNIPAm/GO showed only an 11.7% decrease in linear swelling ratio. Light irradiation of the PNIPAm/rGO(96 h) led to a decrease of the *λ*_*f*_ from 1.20 to 0.70, which corresponds to an approximately 80.1% decrease in the volume compared to the initial equilibrium state. These measurements indicate that the shrinkage of the hydrogels under light illumination increased with the increase in the reduction time, meaning that the photothermal efficiency of GO was significantly enhanced by reduction with hydrazine monohydrate. It means that the photothermal efficiency of GO was significantly enhanced by reduction. As a result, we could maximize the light induced volume shrinkage by reduction of GO dispersed in the hydrogel matrix. We also found that this reversible volume change of the composite hydrogel triggered by visible light could be repeated multiple times without significant variation. (Fig. 2b) Thus, further reduction of the GO by visible-light irradiation did not occur, which might have caused an increase of the light induced volume shrinkage due to the enhanced photothermal efficiency.

To confirm the improved photothermal efficiency by the reduction of GO, the temperature increases under light irradiation in aqueous GO and rGO solutions were examined under the light irradiation. The rGO was prepared by the treatment of GO in 0.33 M aqueous hydrazine monohydrate at RT. As seen in Fig. 2c, the temperature of the aqueous GO solution increased from 24.3 to 31.1 ^o^C after 3 min light exposure. In contrast, the temperature of the aqueous rGO solution with 96 h reduction time increased from 24.4 to 36.8 °C under identical irradiation conditions. These results indicated that the efficiency of a photothermal effect is enhanced through reduction of GO, confirming that the increased light induced volume shrinkage of the PNIPAm/rGO was resulted from the enhanced photothermal effect of rGO. We found that the hydrogel containing rGO shows nearly the same thermal behavior independent of reduction time, indicating that the reduction state of rGO have little influence on the thermo-sensitivity of the composite hydrogels. (Figure S1) We also observed that pore size and network density of the composite hydrogel was not altered even after the reaction with hydrazine monohydrate for 96 h. (Figure S2) It means that the improved light-induced shrinkage of the PNIPAm/rGO can be attributed to the enhanced photothermal efficiency of rGO.

**Figure 3:**
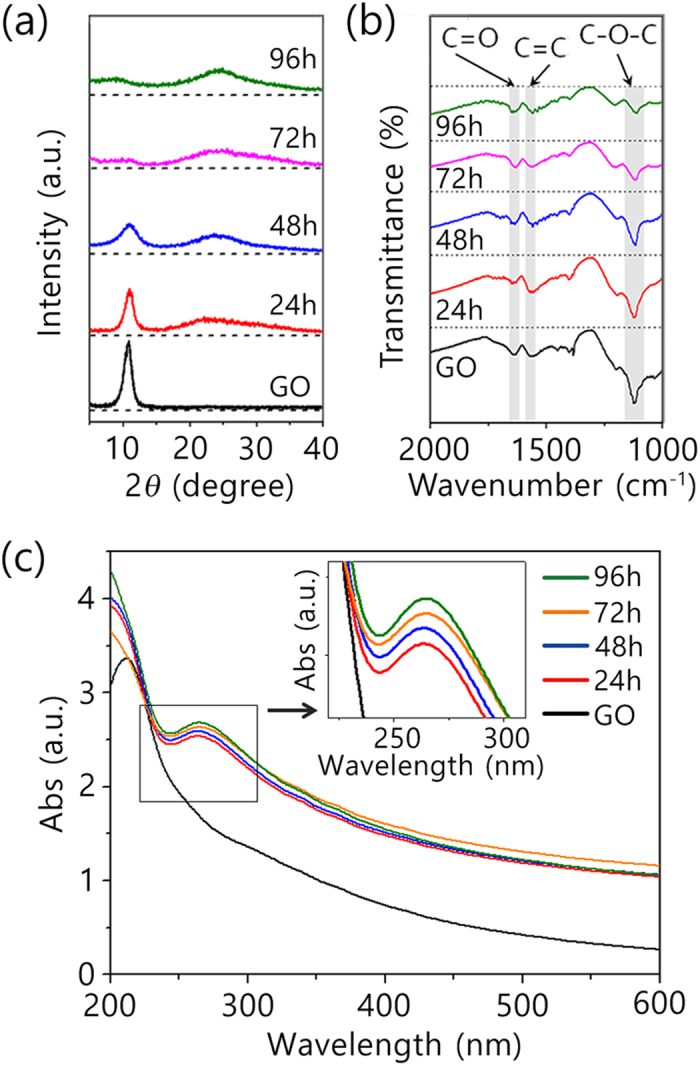
(**a**) XRD profiles, (**b**) FTIR spectra, and (**c**) UV-Vis spectra for GO and rGO after various reduction times.

Next, to investigate the chemical state of the GO before and after soaking in the hydrazine monohydrate, we obtained the XRD profiles and Fourier-transform infrared (FTIR) spectra of a sample treated in 0.33 M aqueous hydrazine monohydrate at RT. We assumed that the chemical environment of aqueous dispersion of GO is quite similar with that of GO dispersed in the hydrogel matrix, since most volume of the hydrogel is occupied by hydrazine solution. Fig. 3a shows the XRD profiles for the dried GO and rGO after various reduction times. The diffraction peak for GO was observed at 2*θ* = 10.8° with a d-spacing of 0.819 nm, corresponding to the (002) reflection of stacked GO sheets. In the XRD profiles of the rGO samples, the intensity of the (002) peak at 10.8° is significantly reduced, although a small bump appears near 23.2° as the reaction time increases. The appearance of the this peak at around 23.2° indicates that the oxygen-containing functional groups attached to the surface of the graphene were removed by reduction^18^.

Figure 3b shows the FTIR spectra for the GO before and after hydrazine treatment. The three significant absorption peaks of GO were obseved at 1115 cm^−1^ due to C-O-C stretching vibration of epoxy group, 1630 cm^−1^ due to C = O of carboxyl group and 1565 cm^−1^ due to C = C skeletal vibration from aromatic domains, indicating the presence of oxygen functionalities in the GO sheets. The spectra of the samples after hydrazine treatment revealed the removal or significant reduction of these characteristic vibrations due to oxygen functionalities. With the increase of reaction time, the strong absorption peak at 1115 cm^−1^ corresponded C-O-C stretching vibration of epoxy group gradually disappears, while the peaks at 1630 cm^−1^ and 1565 cm^−1^ are still observed even after reaction for 96 h. These results were well matched with the XRD profiles, in which the interlayer spacing was gradually decreased due to the reduction of GO. The remaining peaks at 1630 cm^−1^ and 1565 cm^−1^ correspond to the carboxyl functional groups of GO, and indicate their retention even after reaction for 96 h.

Optical properties of the GO before and after hydrazine treatment were also investigated, confirming the change of reduction state of GO. As seen in Fig. 3c, the absorption peak of the GO dispersion at 211 nm suddenly disappeared after treatment with hydrazine monohydrate solution. New absorption peak at 264 nm appeared and the intensity of the peak gradually increase with increase of reduction time, suggesting that the electronic conjugation within the graphene sheets is restored upon reduction. These results were well matched with the XRD profiles and FTIR spectra, confirming that GO sheets were reduced in the hydrazine monohydrate solution at RT.

We can conclude that the GO sheets dispersed in the hydrogel matrix were reduced in the hydrazine monohydrate solution at RT, and the reduction of the oxygenated functional groups results in the enhancement of the photothermal efficiency that induces the large volume shrinkage of the hydrogels triggered by light irradiation.

**Figure 4:**
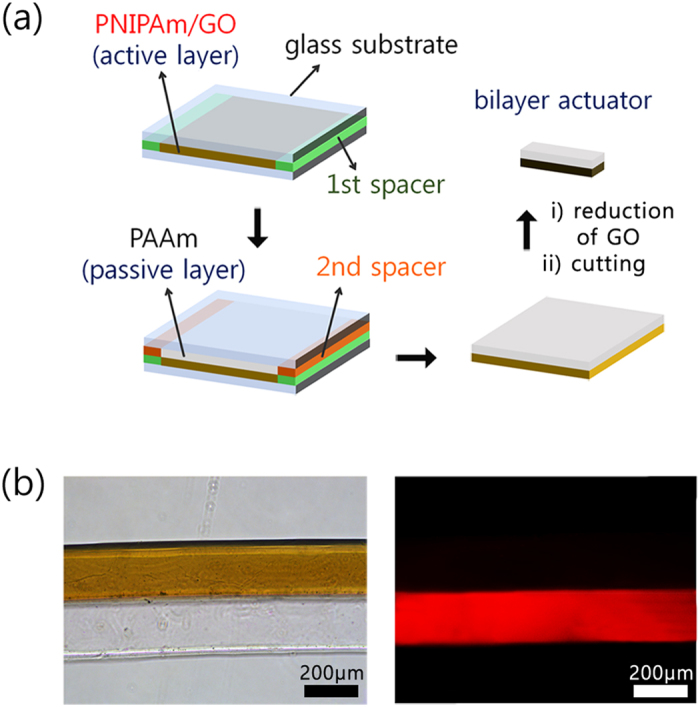
(**a**) Schematic illustration of the layer-by-layer method for the fabrication of a bilayer-type photo-actuator. (**b**) Optical (left) and fluorescence (right) microscope image of fabricated bilayer composed of PNIPAm/GO and poly(acrylamide) (PAAm) incorporating methacryloxyethyl thiocarbonyl rhodamine B monomer layers.

With regard to potential applications of the PNIPAm/rGO composite that shows improved light-induced volume shrinkage, we envisioned a bilayer-type photo-actuator that would achieve a full bending motion upon visible-light exposure. Previous reports demonstrated that the hydrogel bilayer actuators consisted of passive layer and stimuli-responsive active layer show bending motion in response to the environmental changes^3^,^4^,^5^,^6^,^7^,^8^,^9^. In this work, a hydrogel bilayer comprising polyacrylamide (PAAm) as the passive layer and PNIPAm/rGO(96 h) as the active layer was prepared via a layer-by-layer method using radical polymerization, as described in Fig. 4a. First, the PNIPAm/GO layer was prepared by radical polymerization between two glass coverslips separated by 50 μm spacers. After formation of the first hydrogel layer, the top coverslip was removed from the hydrogel sheet, additional spacers were stacked on the existing spacers, and the coverslip was reapplied. Next, the PAAm pre-gel solution was filled in the space above the existing PNIPAm/GO layer. After gelation, the coverslips and spacers were removed from the hydrogel bilayer, and then bilayer was cut to arbitrary dimensions. Using a fluorescence microscope, we confirmed found that no diffusion of the PAAm from the monomer solution to the existing PNIPAm/GO layer occurred (Fig. 4b).

**Figure 5:**
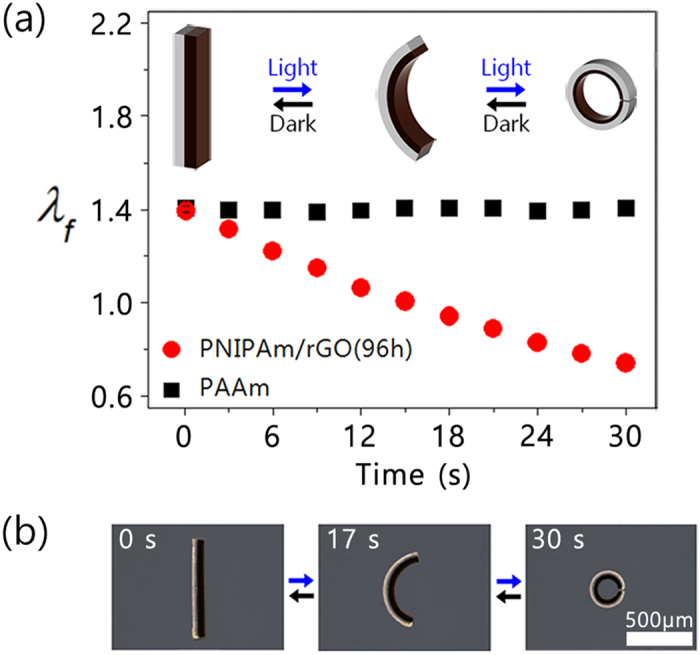
(**a**) *λ*_*f*_ for the active and passive layers of the straight actuator (inset: schematic diagram of bending behavior from stick to arc shape), (**b**) light-induced actuation motion of a straight-type actuator.

The bilayer strips were composed of active and passive layers with similar swelling ratios of 1.39 at equilibrium, leading to the formation of a stick shape, as shown in Fig. 5a. The dimension of the active layer, PNIPAm/rGO(96 h), decreased from 1.39 to 0.74 in response to light irradiation, whereas the passive layer, PAAm, retained its equilibrium dimension. Since the PAAm layer constrains dimensional changes of the PNIPAm/rGO(96 h) layer, the stress at their common boundary induced from size mismatch forced the bilayer to bend one way. The large dimensional difference between two layers induced a large bending motion into a ring via an arc, as illustrated in the inset of Fig. 5a. The light-induced bending motions of the fabricated PAAm@PNIPAm/rGO(96 h) were monitored at 30 °C, and representative images are shown on Fig. 5b. To maximize the shrinkage of the active layer, the medium temperature was set to just below the lower critical solution temperature of the PNIPAm/rGO(96 h)^17^. As expect from the swelling ratios of each layer, the bilayer actuator adopted a stick shape when in the equilibrium state before exposure to light. Exposure to visible light of 41.8 mW/cm^2^ for 30 s induced full bending of the actuator toward the PNIPAm/rGO(96 h) side via an arc shape due to the large shrinkage of the active layer. In addition, the light-induced bending behavior was found to be fully reversible and reproducible. Under identical light irradiation condition, the shape of bilayer actuator comprising PAAm@PNIPAm/GO was not much bent (Figure S3).

**Figure 6:**
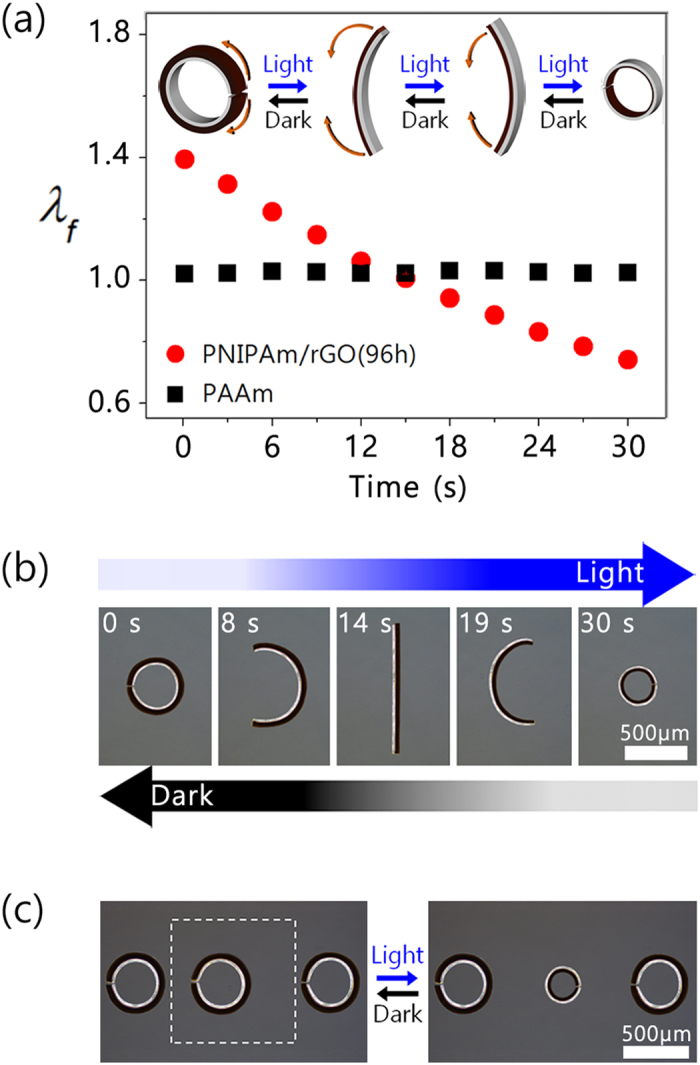
(**a**) λ_*f*_ for the active and passive layers of the circular actuator (inset: schematic diagram of the bending behavior from ring to reverse ring). (**b**) Optical micrographs of the bidirectional bending motion depending on the irradiation time, and (**c**) optical micrographs for the local control of circular actuators by irradiation with blue light in a selected region (dotted square denotes the light irradiation region).

As mentioned above, swelling ratio of the hydrogels can be adjusting by varying crosslinking density, enabling shape and motion variation of the actuators. To obtain a hydrogel photo-actuator with a bidirectional bending ability, the PAAm layer was designed to have a constant swelling ratio of 1.02 by increasing the amount of crosslinker from 0.5 to 3.0 mol%. Thus, the *λ*_*f*_ of PNIPAm/rGO(96 h) layer of 1.39 is greater than that of the PAAm layer, leading to the bending of the actuator in the PAAm direction in the initial state. As the light-induced shrinkage of the PNIPAm/rGO(96 h) increases, the dimensional mismatch between both layers decreases, causing the actuator to unfold. At the time which swelling ratio of both layers matches, the actuator straightens. Further expose to light induces a decrease in the swelling ratio of PNIPAm/rGO(96 h) below that of PAAm, resulting in the bending of the actuator in the opposite direction, toward the PNIPAm/rGO(96 h) layer. As a result, the light-induced shrinkage of the PNIPAm/rGO(96 h) can drive a bidirectional bending motion from a ring to a reversed ring by turning the PAAm layer inside out via the stick shape, as illustrated in the inset of Fig. 6a,b shows the monitored optical micrographs for the fabricated circular actuator under light irradiation. In the initial state, it formed a ring shape bent toward the PAAm side. When exposed to visible light for 14 s, we found that the initially bent bilayer actuator straightened, and then bent fully in the reverse direction in 30 s. These actuator motions were coincident with the measured swelling ratio changes of each layer in response to light irradiation, and also found to be fully reversible and repeatable.

Since the light-irradiated region can be controlled by adjusting of beam size, we attempted the localized control of the hydrogel actuators. As can be seen in Fig. 6c, three bilayer actuators side-by-side in the medium were placed in the medium side by side, and then we examined the light-induced local control of the actuator by irradiating only the center region with visible light for 30 s. Only the actuator in the irradiated region showed a light-driven reverse bending motion, while those in the non-irradiated regions showed no change. This result reveals that the actuation behavior can be controlled independently by local light irradiation.

**Figure 7:**
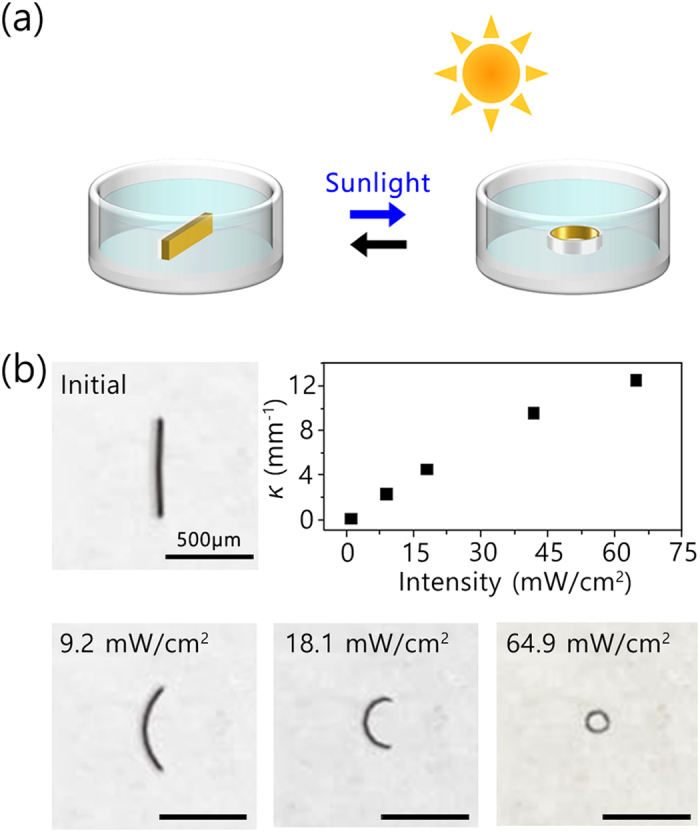
(**a**) Schematic diagram for the sunlight-driven actuation test. (**b**) Photographs of sunlight-driven actuation and curvature (*κ*) changes depending on the intensity.

Motivated by the visible-light-driven actuation of the fabricated hydrogel bilayers, we attempted their control by solar radiation. As illustrated in Fig. 7a, the hydrogel actuator in water at 30 ^o^C was placed outdoors and exposed to sunlight and monitored with digital camera. Fig. 7b shows the bending motion of the bilayer actuator depending on the sunlight intensity. When exposed to sunlight of 9.2 mW/cm^2^ for 5 min, a change in the shape of the actuator was observed from straight to arc. Exposure to stronger sunlight of 64.9 mW/cm^2^ induced further shrinkage of the PNIPAm/rGO(96 h) layer, causing a compressive stress and folding the bilayer to a ring shape. After blocking the sunlight, the ring-shaped actuator recovered its original shape to straight. These results indicate that the bending motion of the bilayer actuator composed of PAAm and PNIPAm/rGO(96 h) could be driven by sunlight irradiation, and that the bending curvatures (*κ*) were determined by the intensity of the sunlight. To the best of our knowledge, this is the first demonstration of a hydrogel actuator whose motion that is driven in response to the sunlight intensity.

## Conclusion

In conclusion, thermally responsive hydrogels containing rGO were successfully prepared by the chemical reduction of GO after their fixation in the hydrogel network. With this approach, we could prepare the light-responsive hydrogels incorporating rGO without any aggregation problem. Significant light-induced shrinkage of the hydrogels could be achieved by the enhanced photothermal efficiency of the rGO incorporated in the hydrogel matrix. Based on the developed light-responsive hydrogels, we could fabricate bilayer-type photo-actuators that exhibited large bending motions triggered by visible light. Adjusting the swelling ratio of the hydrogel layer yielded bidirectional photo-actuators that showed the active motion of turning inside out. Moreover, we demonstrated that the developed hydrogel actuators showed controlled bending motions in proportion to the intensity of the sunlight.

## Methods

### Materials

Aqueous GO sheets (5 mg/mL, composition: carbon (79%); oxygen (20%), flake size: 0.5–5 μm) were obtained from the Graphene Supermarket (Calverton, NY, USA). NIPAm and hydrazine monohydrate were purchased from TCI (Nihonbashi-honcho, Chuo-ku, Japan) and Daejung Chemicals & Metals Co. (Gyeonggi-do, Korea), respectively. Phosphate-buffered saline (PBS) and acrylamide (AAm) were obtained from Bio Basic Inc. (Markham Ontario, Canada). Aqueous fluorescent 3 μm polystyrene beads and methacryloxyethyl thiocarbonyl rhodamine B were purchased from PolyScience Inc. (Warrington, PA, USA). All other chemicals were obtained from Sigma-Aldrich (St. Louis, MO, USA) and used as received.

### Preparation of the composite hydrogels

The PNIPAm/GO hydrogels were prepared by the free radical polymerization of a monomer solution containing the aqueous GO dispersion. The monomer solution (50 μL) containing NIPAm (7.21 mg, 637.2 mM), sodium acrylate (NaAc, 0.19 mg, 19.9 mM), and BisAA (0.10 mg, 6.5 mM) was mixed with the aqueous GO dispersion (50 μL). The concentration of aqueous GO in the mixed solution was 3.3 wt% relative to the monomer content. A small amount (~0.5 μL) of a dilute suspension of fluorescent polystyrene beads was added to the mixed solution before polymerization to facilitate measurement of changes in the linear swelling ratio by the tracking beads using an epi-fluorescent microscope. The concentration of fluorescent beads was selected to yield an average of ~10 to 20 beads in an area of ~1 mm^2^. The polymerization reaction was initiated by adding *N,N,N*′*,N*′-tetramethylenediamine (0.3 μL) and 10 wt% ammonium persulfate aqueous solution (0.6 μL) to the degassed composite solution (100 μL). The resulting solution was immediately inserted through capillary action between two coverslips separated by 140 μm-thick spacers. The gelation process was carried out under nitrogen atmosphere to prevent termination of the free radical polymerization by oxygen. After gelation for 1 h, the coverslips and spacers were removed from the PNIPAm hydrogel composite, and the hydrogel was then swelled in PBS solution (137 mM NaCl). The PBS solution was replaced several times to remove unreacted components over 3 hours.

To reduce the GO dispersed in the hydrogel network, the PNIPAm/GO hydrogel was swelled in 0.33 M hydrazine monohydrate solution. Upon completion of the reaction, the PNIPAm/rGO hydrogel composites were retrieved and then swollen in PBS solution. To remove the remaining reductant from the hydrogel, the swelling medium was changed at least three times.

### Preparation of the hydrogel bilayer actuators

The PAAm@PNIPAm/rGO hydrogel bilayer actuators were prepared using a layer-by-layer method. First, the PNIPAm/GO layer was prepared by radical polymerization between two glass coverslips separated by 50 μm spacers that were made by stacking two polyimide films (Kapton, Dupont). The monomer solution (100 μL) for PNIPAm/GO, with a chemical composition identical to that of the composite hydrogel, was injected into the sample cell. After gelation of the first hydrogel layer, the top coverslip was removed from the hydrogel sheet, additional 25 μm-thick spacers (i.e., the polyimide films) were stacked on the existing spacers, and the coverslip was reapplied. Next, the PAAm aqueous pre-gel solution (100 μL) containing AAm (7.41 mg, 1043.8 mM), BisAA (0.08 mg, 5.2 mM) and methacryloxyethyl thiocarbonyl rhodamine B (0.355 mg, 5.2 mM) was filled in the space above the existing PNIPAm/GO layer, and polymerized for 1 h. After gelation, the coverslips and spacers were removed from the hydrogel bilayer and it was swollen in 0.33 M hydrazine monohydrate solution for 96 h.

The circular PAAm@PNIPAm/rGO bilayer actuator was prepared by an identical procedure, except for the monomer composition and dimensions. First, a 20 μm PNIPAm/GO layer was prepared. The spacers in this case were made by stacking two commercial aluminum foils (SK Aluminum, Korea). After complete polymerization (1 h), the upper coverslip was removed. An extra 10 μm-thick aluminum foil spacer was inserted and the coverslip was reapplied. A degassed PAAm pre-gel solution containing AAm (7.03 mg, 988.8 mM) and BisAA (0.47 mg, 30.6 mM) was inserted. After gelation, the coverslips and spacers were removed, and the hydrogel bilayer was swollen in distilled water. The fabricated bilayer actuator was swollen in 0.33 M hydrazine monohydrate solution for 96 h to reduce the GO dispersed in the PNIPAm. After washing the remaining reductant, the bilayer-type photo-actuator was cut to dimensions of 215 by 760 μm for a straight shape or 220 by 1230 μm for a circular shape.

### Measurements

To assess the extent of reduction during the reaction with hydrazine monohydrate solution, XRD and FTIR spectroscopy were carried out with the GO and rGO powders. The rGO powder was made by mixing the aqueous GO and 0.33 M hydrazine monohydrate solutions. Upon completion of the reduction, the mixture was dropped on a glass substrate and dried to collect the rGO powder. The XRD spectra were recorded from 5 to 40° with Cu K_α_ radiation (Ultima IV, Rigaku). The FTIR spectra were recorded from 400 to 4000 cm^−1^ (Nicolet 380, Thermo Scientific). The UV-Vis spectra of the GO and rGO were measured with a UV-Vis spectrometer (V-550, Jasco Inc.).

To measure the changes in the linear swelling ratio induced by visible-light irradiation, the hydrogel composites were exposed to blue light generated from a high-pressure mercury short arc lamp (EL6000, Leica) through a blue excitation filter (450–490 nm, I3, Leica) with an intensity of 41.8 mW/cm^2^. The changes in the linear swelling ratio were measured by tracking the fluorescent beads within the hydrogel composites using an epi-fluorescent microscope (DMI-3000B, Leica), as described previously^35^. Intensity of blue light and sunlight was measured by an intensity meter (843-R, Newport) with thermophile sensor (919P-003–10, Newport). To demonstrate sunlight-driven actuation, the hydrogel actuator in water at 30 °C was exposed to sunlight. Before being exposed to sunlight, the sample was placed outside in the shade until it reached at equilibrium temperature. The image for initial state was taken immediately after exposure to sunlight, and other images were taken after exposure for 5 min. Intensity of sunlight was varied by exposing the sample at different time of the day.

## Additional Information

**How to cite this article**: Kim, D. *et al.* Highly bendable bilayer-type photo-actuators comprising of reduced graphene oxide dispersed in hydrogels. *Sci. Rep.* **6**, 20921; doi: 10.1038/srep20921 (2016).

## Supplementary Material

Supplementary Information
