# Supplementary material for: Highly bendable bilayer-type photo-actuators comprising of reduced graphene oxide dispersed in hydrogels
Source: Sci Rep. 2016 Feb 11;6:20921. doi: 10.1038/srep20921 (PMC4750031; doi:10.1038/srep20921)
Supplement: Supplementary Information [file srep20921-s1.pdf]

Supplementary Information

Highly bendable bilayer-type photo-actuators comprising of reduced graphene oxide dispersed in hydrogels

Dowan Kim[1], Heon Sang Lee[2], Jinhwan Yoon[1,]*

[1]Department of Chemistry and [2]Department of Chemical Engineering

Dong-A University, 37 Nakdong-Daero 550beon-gil, Saha-gu, Busan 49315, Republic of Korea

Email: jyoon@dau.ac.kr

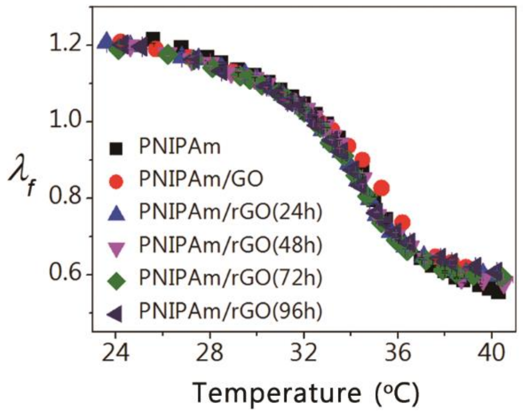

Figure S1. Temperature-dependent linear swelling ratio, λ_f, of PNIPAm/GO and PNIPAm/rGO(*N*).

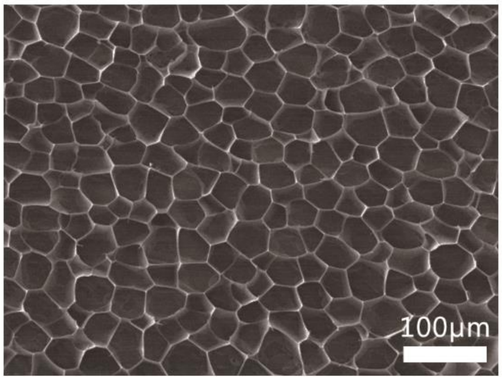

PNIPAm/GO

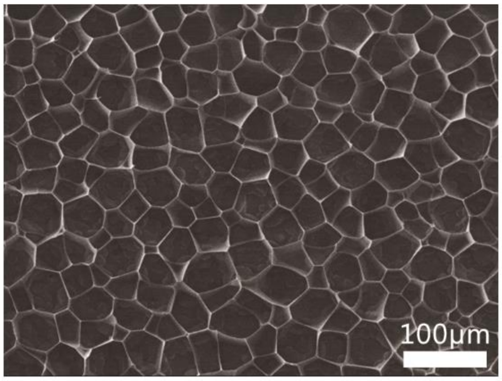

PNIPAm/rGO(96h)

Figure S2. Scanning electron microscopy images of PNIPAm/GO and PNIPAm/rGO(96h). SEM images were obtained using freeze dried samples at 5.0 kV and a working distance of 8.0 mm (JSM-6700F, JEOL, Japan).

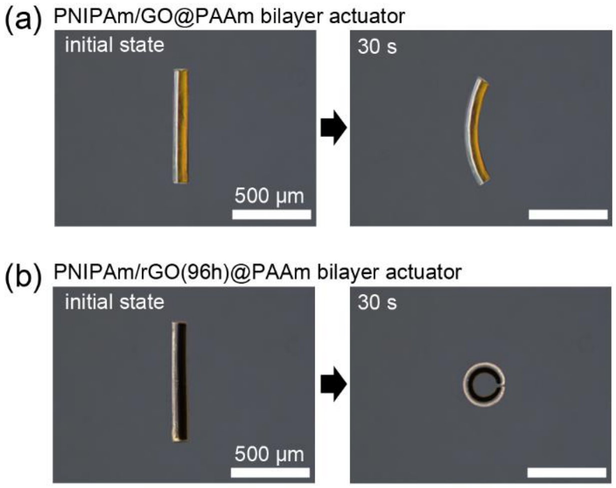

Figure S3. Light-induced actuation motion of (a)PNIPAm/GO@PAAm bilayer actuator and (b)PNIPAm/rGO(96h)@PAAm bilayer actuator.
